# Femtosecond Laser Polishing of AlN Ceramics and Numerical Simulation of Ablated Morphology

**DOI:** 10.3390/mi16121303

**Published:** 2025-11-21

**Authors:** Ruikang Shi, Zhenyu Zhao, Houming Zhou, Jin He

**Affiliations:** 1 College of Sino-German, Shenzhen Institute of Information Technology, Shenzhen 518172, China; 202321642825@smail.xtu.edu.cn (R.S.); 2300291022@email.szu.edu.cn (J.H.); 2 School of Mechanical Engineering and Mechanics, Xiangtan University, Xiangtan 411105, China; 3 School of Mechatronics and Control Engineering, Shenzhen University, Shenzhen 518060, China

**Keywords:** femtosecond laser polishing, aluminum nitride ceramics, response surface methodology, numerical simulation, surface integrity

## Abstract

To meet the surface polishing requirements of aluminum nitride (AlN) ceramics, this study developed a multi-objective optimization experimental model based on response surface methodology (RSM), with surface roughness as the key optimization target. A systematic series of femtosecond laser polishing experiments were conducted. Polishing effectiveness and the evolution of material properties under different process parameters were comprehensively evaluated through surface morphology characterization, microhardness testing, friction and wear experiments, and energy-dispersive X-ray spectroscopy (EDS) analysis. The experimental results indicated that the optimal combination of process parameters, as determined by RSM optimization, was identified as a laser power of 17.43 W, pulse frequency of 292.29 kHz, and scanning speed of 1004.82 mm/s. Under these parameters, femtosecond laser polishing significantly reduced the surface roughness of the AlN ceramic, with the initial Ra value decreasing from 2.513 μm to 0.538 μm, a reduction of 78.57%. Compared to CO_2_ laser polishing (*R*_a_ = 0.817 μm), femtosecond laser polishing demonstrated superior performance in enhancing surface quality. Analysis of the microstructural mechanisms revealed that the femtosecond laser, due to its ultra-short pulse characteristics, effectively suppressed the expansion of the heat-affected zone. It passivated surface microcracks through a photothermal ablation effect and reduced the thickness of the subsurface damage layer. Furthermore, the friction coefficient and wear rate of the polished samples decreased, indicating a significant improvement in wear resistance. On the numerical simulation front, a multi-physics model describing the interaction between the femtosecond laser and AlN ceramic was established based on the non-equilibrium two-temperature model (NTTM) coupled with solid mechanics. The key innovation of our model is the full coupling of heat transfer and solid mechanics, which allows for an accurate revelation of the material morphology evolution mechanism during femtosecond laser ablation. The model’s accuracy is confirmed by the excellent agreement with experimental results, showing relative errors of only 3.23% and 12.5% for the melt pool width and depth, respectively.

## 1. Introduction

Aluminum nitride ceramics are widely recognized as extremely valuable semiconductor substrates and packaging materials due to their excellent properties, including high thermal conductivity, a thermal expansion coefficient close to that of silicon, and low dielectric constant [1,2,3]. In practical applications, the surface roughness at the interface between aluminum nitride ceramic substrates and chips directly affects heat dissipation efficiency and signal transmission performance. Low-damage polishing processes can suppress the formation of microcracks, effectively extending the service life of power modules. However, due to the high thermal conductivity, low fracture toughness, and thermal sensitivity of aluminum nitride ceramics, traditional thermal polishing processes are prone to causing thermal damage and microcrack propagation, leading to degradation of their intrinsic properties [4,5]. Commonly used polishing methods include mechanical polishing, chemical polishing, chemical–mechanical polishing, and fluid polishing, among others. The plasma-assisted polishing technique proposed by Sun et al. [6] is a dry polishing technology. Its principle involves using plasma irradiation to induce surface modification, combined with ultra-low pressure or soft abrasives to remove the modified layer, making it suitable for difficult-to-machine materials such as aluminum nitride. This technology overcomes the limitations of traditional mechanical processing by leveraging a chemical–physical synergistic mechanism, providing a new method for ultra-precision polishing of hard and brittle materials. Zhao [7] et al. employed a polishing solution containing Fenton reagents for magnetorheological polishing of AlN ceramics. The strong oxidative effect of hydroxyl (·OH) radicals promotes the formation of a softened layer on the material surface, increasing material removal rates by approximately 40% compared to traditional processes, while achieving an ultra-smooth surface without mechanical polishing marks. Laser polishing, as a non-contact precision processing technology, induces surface melting and recrystallization or selective vaporization of materials using high-energy laser beams, enabling precise control of micro- and nano-scale surface roughness within sub-second time scales. In recent years, the field of efficient processing of hard and brittle materials has garnered significant attention [8]. Laser polishing technology is typically categorized into two types based on its mechanism of action: thermal polishing and cold polishing [9]. Femtosecond laser cold polishing, based on photochemical ablation mechanisms, enables material removal with minimal thermal influence zones, offering significant advantages in suppressing thermal damage, preserving material intrinsic properties, and enhancing surface quality [10,11,12], making it an ideal polishing technology for aluminum nitride ceramics. Ihlemann et al. [13] compared the mechanisms of nanosecond and femtosecond laser polishing of oxide ceramics. Nanosecond lasers use a plasma-shielded thermal melting mechanism, resulting in a deeper heat-affected zone; femtosecond lasers achieve non-thermal removal through multiphoton absorption and photochemical decomposition, resulting in a smaller heat-affected zone and a reduction in surface roughness of approximately 40%. Taylor et al. [14] first validated the feasibility of femtosecond laser polishing of silicon carbide, finding that excessively high pulse frequencies can lead to thermal accumulation, causing surface overheating and oxidation. Optimizing frequency control to keep temperatures below the threshold can suppress oxidation and improve quality. Chen et al. [15] used a hyperbolic heat conduction model to reveal differences in ablation kinetics between ultrashort pulse lasers and AlN, successfully predicting single-pulse ablation depth after introducing a temperature rise delay effect. Preusch F et al. [16] achieved high-precision and efficient micro-processing of AlN using a near-infrared pulsed fiber laser. By regulating the repetition frequency and pulse overlap rate, they quantified the processing quality and efficiency parameters. The formidable challenge in polishing aluminum nitride (AlN) ceramics stems from an intrinsic thermomechanical dilemma: its high hardness and low fracture toughness predispose it to mechanical damage, whereas its high thermal conductivity and sensitivity often induce thermal damage during conventional laser processing. Femtosecond lasers present a theoretically ideal solution to this dilemma; their ultrashort pulse duration—significantly shorter than the electron–phonon coupling time—enables concurrent suppression of the heat-affected zone and mechanical stresses, paving the way for low-damage polishing. However, the absence of a systematic and quantitative framework elucidating the synergistic effects of critical parameters (e.g., laser power, scanning speed, and pulse repetition rate) on the final surface morphology remains a critical bottleneck. This knowledge gap impedes the transition of femtosecond laser polishing from laboratory-scale validation to stable, reproducible industrial deployment.

This study employed response surface methodology (RSM) to systematically optimize the femtosecond laser polishing process for aluminum nitride (AlN) ceramics. Based on a Box–Behnken design (BBD), three factors—laser power, scanning speed, and pulse frequency—were selected, each with three levels. This design required only 17 experimental runs, efficiently replacing the 27 runs needed for a full factorial design and significantly enhancing experimental efficiency. A quadratic regression model was developed to correlate the key process parameters with the target response, enabling the analysis of multi-parameter interactions and the determination of the optimal process window. To highlight the advantages of the femtosecond laser process, a comparative study was conducted using a carbon dioxide (CO_2_) pulsed laser for thermal polishing. Furthermore, a parametric sweep of the key parameters was performed via COMSOL Multiphysics 6.3 simulations to gain deeper insight into the mechanisms by which the coupled thermo-mechanical fields influence the evolution of surface morphology during polishing.

## 2. Materials and Methods

### 2.1. Experimental Setup and Polishing Experiments

**Figure 1:**
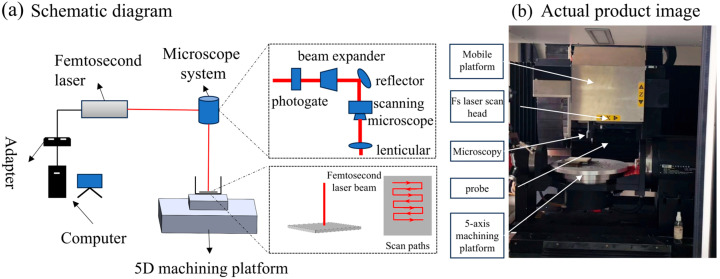
Experimental setup and equipment for polishing AlN.

High-purity aluminum nitride ceramics prepared using a high-temperature, high-pressure sintering process were cut into 100 mm × 100 mm × 5 mm substrates. Their chemical composition and impurity distribution are shown in Table 1. The laser polishing experimental platform was constructed based on a five-axis CNC infrared femtosecond laser processing device (schematic diagram shown in Figure 1a, actual photo shown in Figure 1b). The laser used is the 30 W infrared femtosecond laser (OR-30-IR) developed by Hangzhou Aochuang Photonics Technology Co., Ltd., Hangzhou, China, which provides an average power of 30 W, pulse width ranging from 500 fs to 10 ps, a center wavelength of 1031.8 nm, and a maximum single-pulse output energy of 1.2 mJ. The repetition rate is continuously adjustable from 25 kHz to 1 MHz. Polishing was performed employing a laser with a focal spot diameter of 20 μm, a scanning spacing of 2.5 μm, and a pulse width of 500 fs; this configuration ensured a high overlap between adjacent scanning tracks to facilitate a uniform and smooth polished surface.

Response surface methodology is an experimental design method that integrates mathematical modeling and statistical optimization. In this study, based on the results of a single-factor preliminary experiment, a second-order polynomial model was constructed between key process parameters and target responses to analyze multi-factor interactions and locate the global optimum solution. By constructing a second-order mathematical model of laser energy power, scanning speed, and laser frequency, the multi-parameter interactions were accurately analyzed, and a Box–Behnken design strategy was used to replace the 27 full-factor experiments with 17 experiments. The significance of the model is validated through analysis of variance to ensure the reliability of the optimization. Furthermore, a comparative analysis was conducted with a carbon dioxide (CO_2_) pulsed laser to provide a benchmark against the femtosecond laser polishing technique. The experimental design for the three-level, three-factor response surface methodology (RSM) is outlined in Table 2. The factors—laser power, scanning speed, and pulse frequency—were each set to three levels. The level ranges were determined based on preliminary single-factor experiments, which identified an optimal parameter region near 17.5 W, 1000 mm/s, and 300 kHz. Accordingly, these values were designated as the central levels, and the experimental domain was constructed by establishing appropriate high and low levels around this range to enable a systematic analysis of parameter effects.

### 2.2. Numerical Simulation

#### 2.2.1. Physical Models and Assumptions

Laser processing involves strong nonlinear coupling across multiple physical fields, making it extremely challenging to construct a mathematical model that fully reflects actual operating conditions. To balance the effectiveness of numerical simulation with computational feasibility, the following key assumptions are introduced:

1. The laser beam energy follows a Gaussian spatial distribution, ignoring its distortion and fluctuations;
2. The substrate is treated as a continuous homogeneous medium, ignoring internal microdefects and anisotropy;
3. The processing interface is simplified to an ideal plane, ignoring the influence of surface morphology on energy absorption.

To optimize computational efficiency and reduce resource consumption, a scaled-down geometric modeling strategy was adopted.

**Figure 2:**
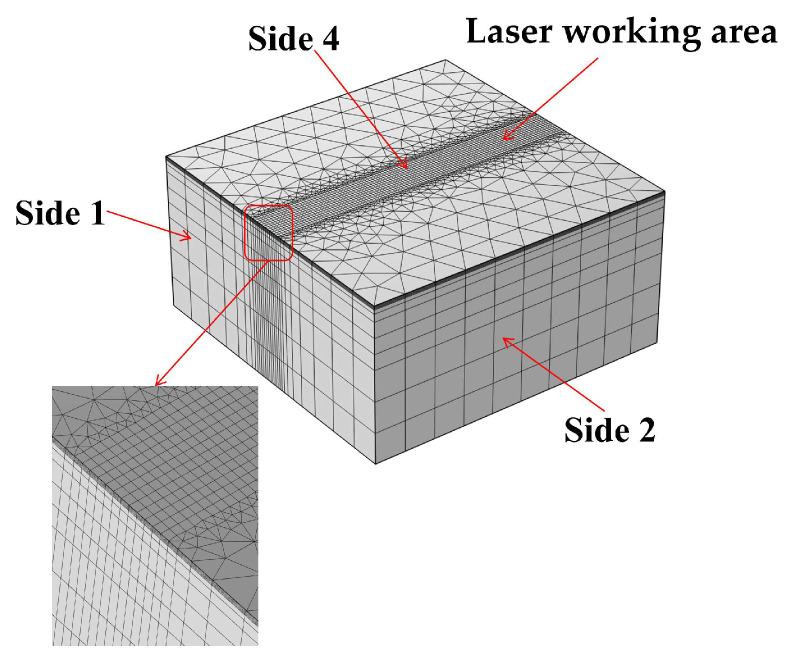
Three-dimensional simulation model.

This method employs a miniaturized simulation model (dimensions: 100 μm × 100 μm × 50 μm, with the corner center point at (−20, −50, 25), see Figure 2) to significantly reduce the number of finite element grids and iteration calculations while retaining key physical characteristics. To accurately track the evolution of temperature and stress on the laser-polished surface, the model employs a free-form triangular mesh with extremely fine mesh sizes in the illuminated region. Applying coarser mesh resolutions to non-critical regions significantly reduces computational requirements.

#### 2.2.2. Setting of Laser Heat Source

During the interaction between femtosecond lasers and materials, photon energy is preferentially absorbed by free electrons. Since the characteristic time of the electron–phonon coupling process is approximately picosecond-scale, the lattice thermal relaxation effect can be neglected during the femtosecond pulse interaction, and only the photoexcitation dynamics of photo-excited electrons need to be considered [17]. This study qualitatively investigated the interaction mechanism between femtosecond lasers and aluminum nitride ceramics based on a three-dimensional dual-temperature theoretical framework. The theoretical model can be characterized by the following set of coupled nonlinear differential equations [18], electronic temperature evolution equation:

(1) $$C_{e}\left(T_{e}\right)\frac{\partial T_{e}}{\partial t}=\nabla \cdot \left(k_{e}\nabla T_{e}\right)-G\left(T_{e}-T_{l}\right)+S\left(z,t\right)$$

where $C_{e}$ is the electron specific heat capacity, $k_{e}$ is the electron thermal conductivity, and G is the electron–phonon coupling coefficient. The electron temperature ($T_{e}$) and the lattice temperature ($T_{l}$) describe the transient thermal states of the two subsystems, g is the coupling coefficient describing the efficiency of the electron–phonon energy transfer, and $S\left(z,t\right)$ is the incident laser energy density function characterized by a space-time Gaussian distribution.

The electron–lattice coupling factor, G, a critical parameter determining the coupling time, is a function of the temperatures of the electron and lattice subsystems. However, it is often treated as a constant in many studies. For a more accurate simulation, the present model adopts the temperature-dependent formulation of G proposed by J. K. Chen et al. [19]:

(2) $$G\left(T_{e},T_{1}\right)=G\left[\left(\frac{A_{e}}{B_{l}}\right)\left(T_{e}+T_{1}\right)+1\right]$$

where *G* denotes the electron–lattice coupling factor at room temperature, $A_{e}$ and $B_{l}$ are material-specific parameters, and $T_{e}$ and $T_{1}$ represent the electron and lattice temperatures., respectively.

Lattice Temperature Evolution Equation:

(3) $$C_{l}\left(T_{l}\right)\frac{\partial T_{l}}{\partial t}=\nabla \cdot \left(k_{l}\nabla T_{l}\right)+G\left(T_{e}-T_{l}\right)-\frac{\partial Q}{\partial t}$$

where $C_{l}$ is the lattice-specific heat capacity, $k_{l}$ is the lattice thermal conductivity, and Q is the latent heat of phase change.

Energy deposition term (laser–material interaction):

(4) $$S\left(z,t\right)=\left(1-R\right)\alpha I_{0}e^{-\alpha z}\cdot exp\left(-\frac{t}{\tau }\right)$$

where *R* is the reflectivity, α is the absorption coefficient, *τ* is the laser pulse width, and $I_{0}$ is the peak intensity.

Laser heat source and convective boundary (Side 4): Spatio-temporally correlated laser heat flux is applied at the Side 4 boundary as the primary heat input.

(5) $$q_{\mathrm{laser}}(r,t)=M(t)\cdot C(t)\cdot \frac{2F}{t_{p}}exp\left(-2\frac{(x-v_{1}t{)}^{2}+y^{2}}{r_{0}^{2}}\right)$$

where F is the laser single pulse energy; $t_{p}$ is the laser pulse width; $v_{1}$ is the laser scanning speed; *r*_0_ is the laser spot radius; M(t) is the function characterizing the laser pulse time envelope; and C(t) is the laser scanning working cycle function. This heat source term will be superimposed on the convection term describing the electron–lattice interaction in the electron heat transfer equation.

The remaining boundary constraints are imposed, as follows:

(6) $$C_{e}\cdot {\left.\frac{\partial T_{e}}{\partial t}\right|}_{x}=C_{1}\cdot {\left.\frac{\partial T_{1}}{\partial t}\right|}_{x}=0$$

During the interaction between femtosecond laser and aluminum nitride, the transient density of free electrons increases dramatically, which influences subsequent processes. Therefore, it is essential to accurately determine this density. The Fokker–Planck equation was employed to simulate free electron density (ne), incorporating mechanisms such as multiphoton absorption, carrier diffusion, and Auger recombination [20,21].

(7) $$\frac{\partial n_{e}}{\partial t}=\nabla \left(D\nabla n_{e}\right)+\alpha_{i}In_{e}+\frac{\delta_{N}}{N\hbar \omega }I^{N}-\frac{n_{e}}{\tau }$$

where I is the laser intensity, τ is the free electron decay constant, and $\alpha_{i}$ stands for the impact ionization coefficient; the impact ionization coefficient αᵢ is derived from electron avalanche ionization theory. Within the framework of the Drude model, it incorporates both hot electron distribution and interband transition probabilities. $\delta_{N}$ is the N-photon cross-section coefficient; for the AIN studied in this work (bandgap ~6.2 eV) and the 1031.8 nm laser (photon energy ~1.201 eV), the theoretical minimum value of N is 7, and ℏω is the photon energy. The diffusion coefficient D is defined as D = *k_B_T_e_u_e_/e*, where *k_B_* and *T_e_* stand for the Boltzmann constant and the electron temperature, and *u_e_* and *e* represent the electron charge and electron mobility. The number of photons required to free a bound electron is determined by the ratio of the material bandgap to the photon energy [22,23]. In this study, the seven-photon absorption of AlN was considered, given that the laser wavelength used was 1031.8 nm (1.201 eV); 7 ×E_photon ≈ 8.4 eV > 6.2 eV.

Upon exposure to femtosecond laser pulses, the AlN material becomes highly excited, resulting in a significant increase in the transient density of free electrons. The optical properties of the material undergo transformation as a consequence. According to the Drude model, the dielectric function is defined by the following expression [24]:

(8) $$\varepsilon =\varepsilon_{1}+i\varepsilon_{2}=1+\left(\frac{n_{e}e^{2}}{m_{e}\varepsilon_{0}}\right)\left(\frac{-\tau_{e}^{2}+i\tau_{e}/\omega }{1+\omega^{2}\tau_{e}^{2}}\right)$$

where ε represents the complex dielectric function, while $\varepsilon_{1}$ and $i\varepsilon_{2}$ represent its real and imaginary components, respectively; $m_{e}$ represents the mass of an electron; ω stands for the laser frequency; and $\tau_{e}$ denotes the free electron relaxation time.

#### 2.2.3. Stress Field Setup

In the pulsed laser surface treatment process, the dynamic response of the temperature field under thermal loading can be synchronously tracked and the mechanism of the material thermoelastic parameters on the characteristics of the thermo-mechanical stress distribution can be revealed through the introduction of a multi-physical field coupling framework in solid mechanics. The ontological relationship of this multi-field coupled process can be characterized by the following mathematical model [25]:

(9) $$\rho \frac{\partial^{2}u}{\partial t^{2}}=\nabla \cdot \sigma +F_{V}$$

where u is the displacement field; ∇ is the gradient operator; σ is the Cauchy stress tensor; and $F_{V}$ is the force per unit deformed volume. Table 3 shows the numerical simulation parameters of femtosecond laser polishing of aluminum nitride ceramic materials.

## 3. Results and Discussion

### 3.1. Construction of a Surface Roughness Prediction Model Using the Response Surface Method

Based on Box–Behnken experimental data, a response surface model for surface roughness was constructed using Design Expert. The significance of the model was evaluated using analysis of variance, and the multiple correlation coefficient and model reliability were calculated. The modeling results are shown in Table 4. Variable definitions: A is laser power, B is scanning speed, and C is pulse frequency; the interaction term between A and B (power × speed) is defined similarly for AC and BC.

The results of the analysis of variance (Table 5) show that the response surface model has high statistical significance in fitting surface roughness, explaining 98.19% of the variation in response values. The model passed the misfit test, demonstrating reliable predictive performance, and the difference between R^2^_adj_ and R^2^_pre_ (0.0858) is less than the critical threshold of 0.2, indicating that the model is not overfitted. The influence patterns of each factor are as follows: the quadratic terms of laser power and scanning speed have a highly significant effect on roughness, contributing 67.54% and 32.94%, respectively; the interaction between laser power and scanning speed is significantly significant, indicating that their synergistic effect requires focused regulation.

The following is a strictly corrected quadratic polynomial regression model equation based on ANOVA table data:

*Ra* = 0.5363 + 0.0191×A + 7.9999 × 10^−4^×B + 0.0253×C + 0.015×AB + 2.5 × 10^−3^×AC−2.499 × 10^−3^×BC + 0.0812×A^2^ + 0.0567×B^2^ + 0.0372×C^2^.

**Figure 3:**
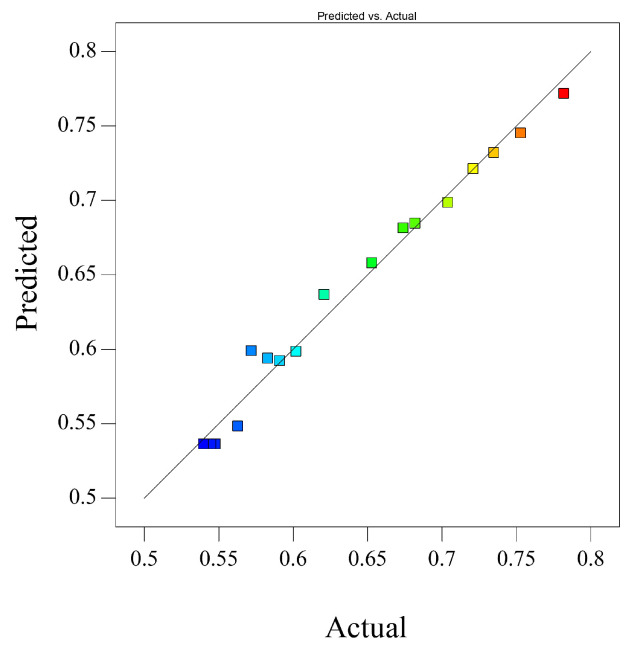
Comparison between model-predicted and actual value.

As can be seen from the comparison of actual values and predicted values (Figure 3), the data points are evenly distributed on both sides of the fitted straight line, indicating that the response surface model constructed is reasonable. The optimal process parameter combination optimized based on this model is *p* = 17.43 W, v = 1004.82 mm/s, f = 292.29 kHz. Under these conditions, the laser fluence is approximately 0.416 J/cm^2^, and the transverse laser spot overlap ratio is about 87.5%.

### 3.2. Mechanism of Influence of Parameter Interaction on Surface Morphology

**Figure 4:**
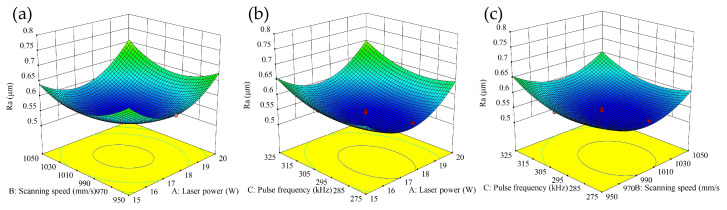
Significant interactions between various parameters on surface roughness. (**a**) Laser power × scanning speed; (**b**) Laser power × laser frequency; (**c**) Laser frequency × scanning speed.

To elucidate the synergistic effects of process parameters on surface roughness during femtosecond laser polishing, a three-dimensional response surface (Figure 4) was constructed based on a quadratic polynomial regression model, visualizing the relationship between parameter coupling and surface roughness. An analysis of variance (ANOVA, Table 5) was first conducted to quantitatively assess the significance of each model term. The model itself was highly significant (*p* < 0.0001) with an *F*-value of 42.18. Among the individual factors, the quadratic term of laser power (A^2^) exhibited a dominant influence (*F*-value = 67.54, *p* < 0.0001), quantitatively confirming a strong nonlinear relationship between laser power and surface roughness and indicating the existence of a distinct optimal power level. This finding aligns with the pronounced curvature observed along the power axis in the response surface (Figure 4). The linear and quadratic terms of pulse frequency (C and C^2^) were also significant (*F*-value = 24.45, *p* = 0.0017; *F*-value = 14.19, *p* = 0.0070, respectively). Notably, while the linear effect of scanning speed (B) was insignificant (*p* = 0.8802), its quadratic term (B^2^) was highly significant (*F*-value = 32.94, *p* = 0.0007), indicating that the influence of speed is also inherently nonlinear. Regarding interaction effects, the power–speed interaction (AB) was the only significant term (*p* = 0.0343), providing a statistical basis for optimizing their combination.

Building upon this quantitative analysis, the response surface interpretation reveals that the strong quadratic effects of power (A) and speed (B), coupled with their significant interaction, enable the ‘moderate laser power and moderate scanning speed’ combination to achieve an optimal balance between energy input and material removal, thereby yielding the minimum surface roughness. Similarly, the synergy between moderate power and moderate pulse frequency effectively suppresses heat accumulation by precisely controlling the thermal input. Conversely, combinations deviating from the optimal range, such as low power with high speed, result in insufficient energy input and inadequate material removal, whereas high power with low speed introduces excessive energy, leading to an expanded heat-affected zone. In both scenarios, the parameters stray from the optimal region of the quadratic response surface, causing a sharp increase in the *Ra* value—a behavior entirely consistent with that predicted by the significant A^2^ and B^2^ terms in the model.

In addition, the study also identified a weak interaction region (Figure 4c). Within this region, the interaction between scanning speed and laser frequency has little influence on surface roughness, and the response surface is relatively flat, indicating that the interaction effect within the range of this parameter combination is not significant. This result is consistent with the statistical conclusions in the analysis of variance (ANOVA) table (Table 5), which further verifies the reliability of the model.

### 3.3. Characterization of Polished Surface Features and Analysis of Performance Evolution

**Figure 5:**
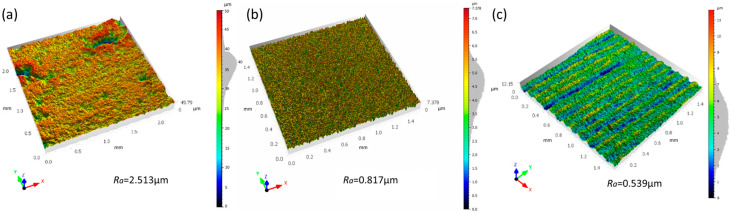
Laser confocal microscopy images of the microscopic morphology of the sample surface. (**a**) Original surface; (**b**) Surface after CO_2_ laser polishing; (**c**) Surface after femtosecond laser polishing.

After processing the sample surface using the optimal parameter combination optimized based on the response surface method, the surface roughness was measured using a confocal microscope (Figure 5). The results showed that the surface roughness values after CO_2_ laser polishing and femtosecond laser polishing were 0.8165 μm and 0.5386 μm, respectively. Compared to the original surface (2.513 μm), femtosecond laser polishing significantly reduced the roughness by 78.57%; however, CO_2_ laser polishing was limited by the thermal melting mechanism, resulting in a limited reduction.

The achieved surface roughness (*Ra* ≈ 0.54 μm) signifies a substantial improvement over the initial state and holds direct engineering relevance. This outcome is of particular importance for high-performance aluminum nitride ceramic substrates in packaging applications, where a surface roughness below 1 μm is typically mandated to ensure effective thermal contact and signal transmission integrity. Our results fully satisfy this critical technical requirement, thereby validating the practical feasibility of the femtosecond laser polishing technique. Furthermore, the process demonstrated exceptional statistical repeatability. To rigorously verify this, three replicate experiments were conducted at the center point of the process parameter space, employing medium levels of laser power, scanning speed, and pulse frequency. The measured surface roughness values were 0.548 μm, 0.545 μm, and 0.541 μm, respectively. This dataset exhibits an exceptionally high degree of concentration, with a standard deviation as low as 0.0035 μm, unequivocally proving the outstanding stability and repeatability of the optimized polishing process.

The predictive capability of the response surface model was further validated by the strong correlation between predicted and experimental values, as illustrated in the scatter plot of Figure 3. The data points are clustered tightly around the 45° diagonal line, indicating a high degree of agreement and confirming the model’s accuracy. This close alignment demonstrates that the second-order polynomial model, developed based on the Box–Behnken design, reliably captures the genuine effects of laser power, scanning speed, and pulse frequency on surface roughness. The robustness of the model provides a solid foundation for the identified optimal process window, ensuring its validity and practical utility.

**Figure 6:**
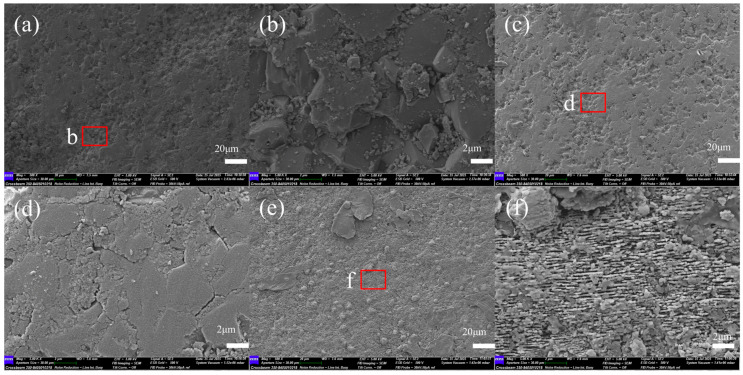
SEM images comparing the surface morphology before and after laser polishing. (**a**) Original surface; (**b**) local magnification of region b; (**c**) CO_2_ laser-polished surface; (**d**) local magnification of region d; (**e**) femtosecond laser-polished surface; (**f**) local magnification of region f.

**Figure 10:**
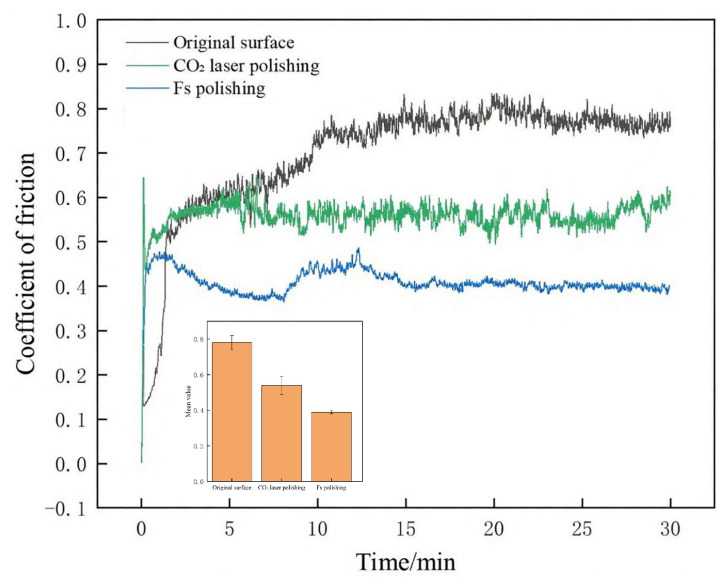
Two-dimensional diagram of friction and wear coefficients.

By examining the surface morphology of the specimen at 500× and 5000× magnification using a scanning electron microscope (Figure 6), a comparison of the microstructural features before and after polishing reveals that while CO_2_ laser polishing improves surface flatness, it also induces noticeable microcracks (Figure 6d). This phenomenon is attributed to the combined effects of laser thermal stress and cooling-induced phase transformation stress exceeding the material’s load-bearing limit. Femtosecond laser polishing (Figure 6e) achieves near-zero thermal damage processing, with surface microcracks largely eliminated; local magnification (Figure 6f) reveals uniformly distributed submicron-scale striations, resulting from the cold processing mechanism of ultra-short pulses suppressing thermal diffusion, significantly reducing the thickness of the subsurface damage layer and microcrack density. Wear performance (see Figure 10) is improved simultaneously.

**Figure 7:**
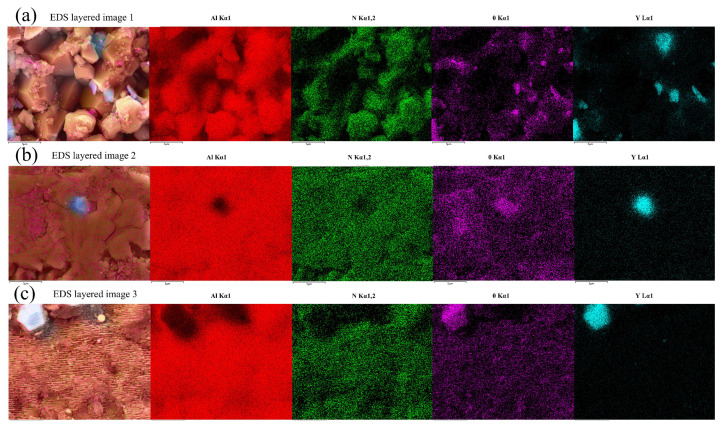
EDS element distribution on the aluminum nitride surface. (**a**) original surface; (**b**) CO_2_ laser polished surface; (**c**) femtosecond laser polished surface.

To investigate the effect of polishing processes on the elemental composition of material surfaces, energy dispersive spectroscopy (EDS) was used to perform surface scans and line scans on the original surface and the polished surface (5000× magnification), comparing the distribution characteristics and relative content differences in elements. The results in Figure 7 show that Al, O, N, and Y elements are uniformly distributed on the original surface and all polished surfaces, confirming that laser polishing did not alter the chemical composition of the material surface.

**Figure 8:**
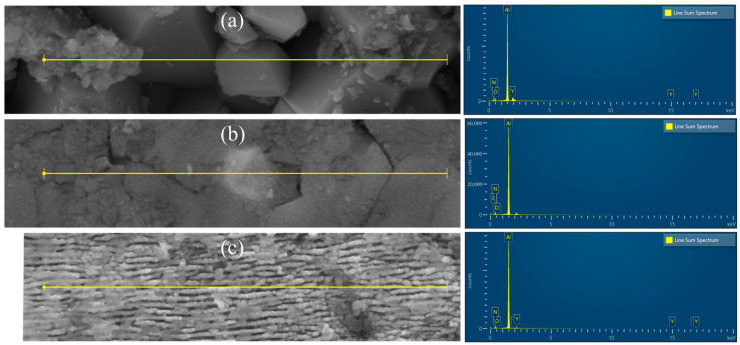
Aluminum nitride wire EDS element distribution. (**a**) Original surface; (**b**) CO_2_ laser polished surface; (**c**) femtosecond laser polished surface.

Line scan analysis (Figure 8) shows that the core elements Al, N, and O exhibit continuous and stable distribution on both the original and polished AlN surfaces, indicating that laser polishing did not disrupt the material’s chemical stability. Among these, CO_2_ laser polishing induced oxidation due to thermal effects, increasing the O element content to 9.73%, while femtosecond laser polishing, due to its cold processing characteristics, suppressed oxidation, resulting in an O element content of 9.13%.

**Figure 9:**
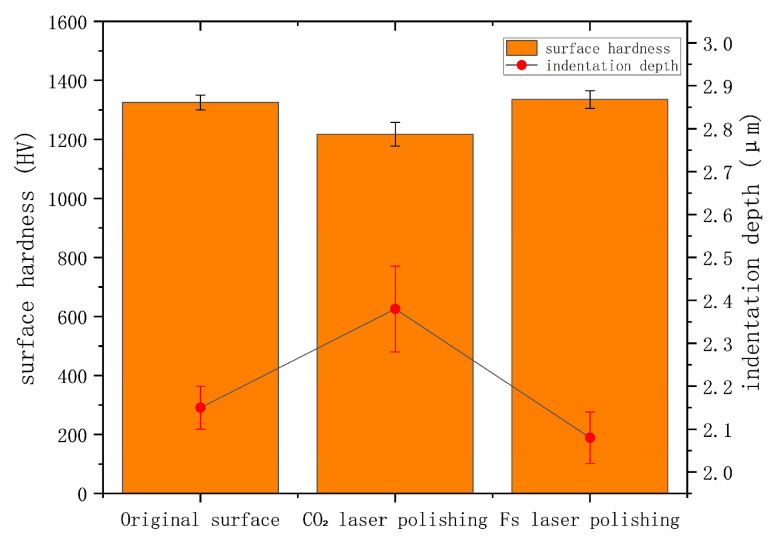
Bar chart of Vickers hardness value distribution and dot–line graph of indentation diameter.

Figure 9 shows the change in surface hardness before and after laser polishing (Vickers hardness tester, load 0.3 kg, average of three tests). The average hardness of the original surface was 1325 HV; after CO_2_ laser polishing, it decreased to 1217 HV, mainly due to the formation of a heat-affected zone, which increased the density of microcracks. Femtosecond laser polishing avoided thermal effects due to its cold polishing mechanism, and the hardness was basically the same as that of the original surface.

To investigate the effect of laser polishing on surface tribological properties, friction and wear experiments were conducted on three surfaces: the original surface, the CO_2_ laser-polished surface, and the femtosecond laser-polished surface. The experiments were performed using a GF-I type friction and wear tester, with a GCr15 bearing steel ball (diameter 4 mm), under a load of 30 N and a speed of 300 r/min for 30 min.

Figure 10 shows that the trends in friction coefficient changes for the three AlN ceramic surfaces differ significantly. The average value for the original surface reached 0.781; after CO_2_ laser polishing, it decreased to 0.543; and after femtosecond laser polishing, it further decreased to 0.389. Analysis indicates that laser processing can improve friction performance: the original surface, due to high roughness, caused micro-asperity fracture and subsurface crack propagation, resulting in significant fluctuations in the friction curve; the CO_2_ polished layer still exhibited fluctuations in the coefficient of friction due to thermally induced oxidation cracks and peeling of the damaged layer; femtosecond polishing formed a nanoscale dense surface through cold processing, stabilizing the coefficient of friction in the 0.38–0.4 range.

### 3.4. Simulation Results and Analysis

**Figure 11:**
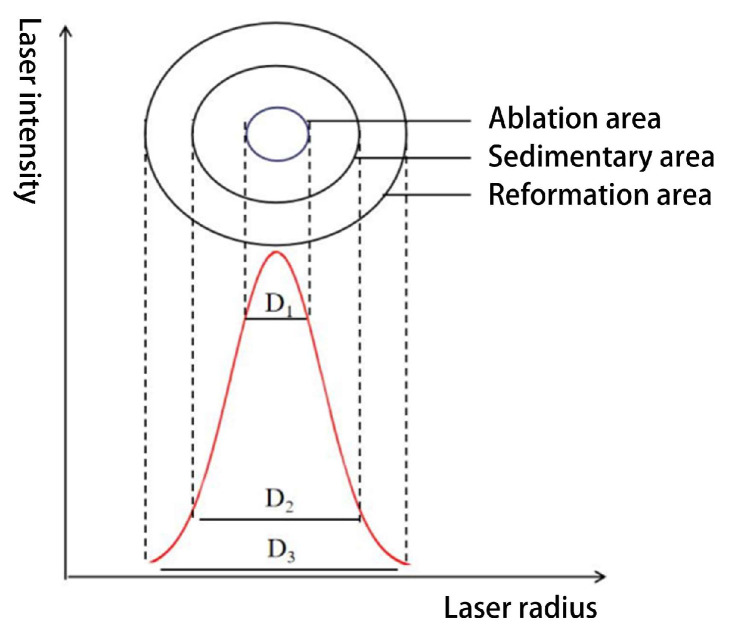
Femtosecond laser spot energy distribution.

Given the characteristics of Gaussian beam energy distribution during material processing, the laser-irradiated region can be categorized into three structural domains: the primary vaporization domain, secondary deposition domain, and material modification domain (as shown in Figure 11), with their respective characteristic diameters being D_1_, D_2_, and D_3_. This layered model reveals that the energy gradient distribution governs the morphological evolution process via a cascading mechanism.

**Figure 12:**
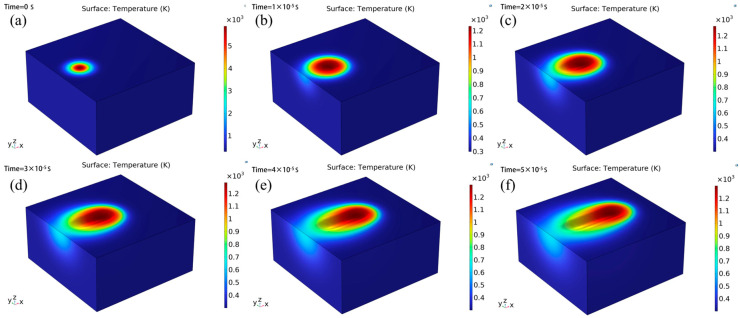
Surface morphology and temperature distribution in the xy plane at the same time: (**a**) t = 0µs; (**b**) t = 10µs; (**c**) t = 20µs; (**d**) t = 30µs; (**e**) t = 40 µs; (**f**) t = 50 µs.

A 3D COMSOL simulation was conducted to analyze the ablation dynamics under the given laser parameters (F = 0.4 J/cm^2^; v = 1004.82 mm/s; f = 292.29 kHz; tp = 500 fs), where the maximum peak power density is calculated as 0.4 / (5 × 10^−13^) = 8 × 10^11^ W/cm^2^. The output time steps were set to range (0, 5, 50) μs. Figure 12 illustrates the evolution of surface morphology and temperature distribution on the material surface at these different time steps. Peak material removal occurs at the center of the Gaussian beam, while the radial energy attenuation generates a parabolic cross-sectional profile. This result is consistent with the expected Gaussian energy distribution pattern and vaporization theory (see Figure 12).

**Figure 13:**
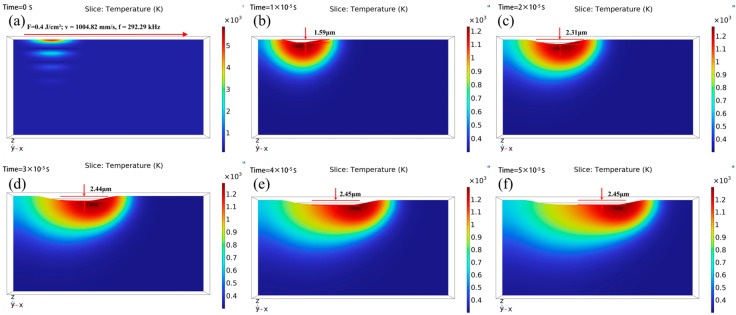
Surface morphology and temperature distribution of the zx center cross-section are shown simultaneously: (**a**) t = 0 µs; (**b**) t = 10 µs; (**c**) t = 20 µs; (**d**) t = 30 µs; (**e**) t = 40 µs; (**f**) t = 50 µs.

By analyzing the material removal process through the zx center section (Figure 13), the simulated polishing depth reached a maximum of 2.45 μm at 4 μs. It exhibits a steady and gradual upward trend.

**Figure 14:**
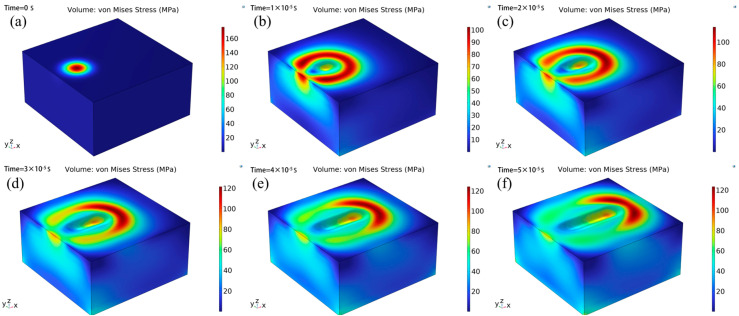
Surface pressure change diagram at different times. (**a**) t = 0 μs; (**b**) t = 10 μs; (**c**) t = 20 μs; (**d**) t = 30 μs; (**e**) t = 40 μs; (**f**) t = 50 μs.

Figure 14 illustrates the evolution of surface stress on the material at different time points. In the initial state (Figure 14a), localized high-stress concentration zones form on the material surface, distributed in a nearly circular pattern, reflecting the localized deposition effect of the initial energy input. During the wavefront expansion and morphological evolution stage (Figure 14b–d), the stress wave spreads outward from the initial concentration zone, with the wavefront morphology rapidly transitioning from a point concentration to a ring-shaped structure, and the spatial extent of the ring-shaped structure expanding over time, demonstrating the spatial diffusion characteristics of the stress wave. Concurrently, the range of the central high-stress zone decreases, and the color scale transitions to medium- and low-stress regions, indicating that stress gradually attenuates as the propagation distance increases, Energy gradually dissipates. Entering the quasi-steady-state diffusion and homogenization stage (Figure 14e,f), the ring-shaped structure continues to expand and tends toward “ellipsoidal symmetry,” the stress peak further decreases, the proportion of medium-stress regions increases, and the stress distribution within the material gradually homogenizes. In the later stage, the stress wave diffusion range approaches most of the cube’s area, and the stress decay rate slows down, reflecting that the thermal–mechanical coupling within the material tends toward a quasi-steady state, with energy dissipation and diffusion reaching equilibrium.

**Figure 15:**
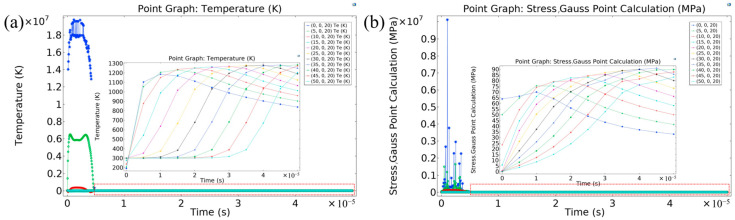
Variations in temperature and stress at the 3D intersection point over different times. (**a**) Temperature variation; (**b**) stress variation.

To track the evolution of temperature and stress along the polishing trajectory, eleven monitoring points were defined along a custom path (start: (0, 0, 20) μm; end: (50, 0, 20) μm; step size: 5 μm) (Figure 15). Simulation results indicate that the temperature evolution at all points exhibits a rapid rise followed by a gradual decline. Starting from an initial temperature of approximately 300 K, the temperature of the electron subsystem surges to a peak of around 2 × 10^7^ K during the initial energy deposition stage, signifying the dominance of a plasma-mediated non-thermal process. Subsequently, via electron–phonon coupling and thermal diffusion, energy is transferred to the lattice and homogenized, shifting the dominant mechanism to thermal ablation. The surface stress evolution synchronizes with the temperature, showing an abrupt initial increase to its peak value, followed by relaxation to below 90 MPa.

The observed phenomena can be explained by the nonequilibrium physics underlying femtosecond laser–matter interactions. First, the extremely high peak temperature originates from the initial nonequilibrium energy absorption process. On the femtosecond timescale, laser energy is preferentially absorbed by the electron subsystem, causing its temperature to rise abruptly. Due to the finite characteristic time of electron–phonon coupling, energy transfer to the lattice remains inefficient at this stage, resulting in a non-thermal state where electrons are extremely hot while the lattice remains relatively cold. Therefore, this peak temperature accurately characterizes the transient thermodynamic state of the electron gas within the energy deposition region and serves as the direct driving force for rapid material ionization and plasma formation. Second, the substantial peak stress is an inherent mechanical response to the aforementioned electronic excitation. Its generation involves two primary mechanisms: the intensely heated electron gas produces enormous thermal pressure, whose rapid expansion launches a strong pressure wave into the material; subsequently, the ensuing thermal ablation process exerts significant recoil pressure on the underlying material, which is further amplified by the inertial confinement of the surrounding material. The peak stress captured in the simulation reflects precisely this transient mechanical response—dominated by electron pressure and modulated by the ablation process—and its magnitude is consistent with theoretically predicted stress levels induced by ultrafast laser-driven shock waves.

**Figure 16:**
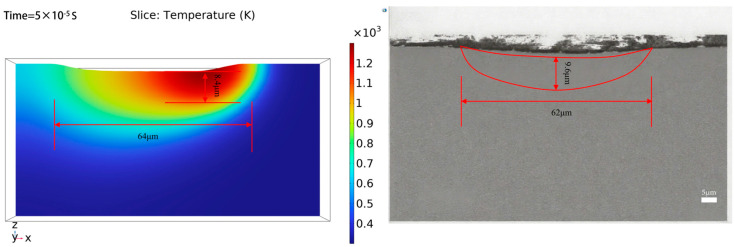
Comparison of the simulated width and depth of the molten pool on the polished surface with the cross-section of the remelted zone on the surface of the laser polishing experiment.

Figure 16 shows a comparison between the simulated melt pool and experimental results. The simulated melt pool width and depth were 64 μm and 8.4 μm, respectively. The solidification morphology of the cross-section (smooth surface sample) was measured using a scanning electron microscope (model: ZEISS GeminiSEM). The experimentally measured melt pool width and depth were 62 μm and 9.6 μm, respectively. The relative errors between the simulated and experimental results for melt pool width and depth were 3.23% and 12.5%, respectively. The simulated molten pool morphology shows good agreement with experimental observations, with a relative error of 3.23% in width and 12.5% in depth. The greater deviation in depth prediction can be attributed to several factors: First, while the pool width is primarily governed by lateral heat conduction, the depth is more susceptible to vertical energy penetration. During actual processing, effects such as multiple reflections within the vapor plume can lead to energy deposition deeper than predicted by the model. Second, the current model does not explicitly account for fluid dynamics within the molten pool—effects driven by Marangoni convection and vapor recoil pressure displace molten material downward, thereby increasing pool depth. Furthermore, as indicated by the temperature contours, the solid–liquid interface at the bottom of the molten pool is diffuse, introducing greater inherent uncertainty in depth determination. Finally, vertical heat conduction is highly sensitive to the temperature-dependent thermophysical properties of materials near the vaporization point; even minor uncertainties in these parameters can be amplified in depth predictions. Despite these simplifications, the relatively low error in depth prediction confirms the model’s utility for understanding the femtosecond laser polishing process.

## 4. Conclusions

This study employs a Box–Behnken design experiment based on response surface methodology to investigate the application of femtosecond laser polishing for ultra-precision machining of aluminum nitride ceramics. The surface roughness is reduced from an initial value of 2.513 μm to 0.5386 μm, representing a relative decrease of 78.57%. Analysis of the response surface results reveals that the interaction between laser power and scanning speed dominates surface morphology optimization, while the synergistic effect between pulse frequency and scanning speed is relatively weak. Compared to CO_2_ laser polishing, femtosecond laser polishing significantly suppresses the expansion of the heat-affected zone, reduces micro-defect passivation and subsurface damage layers, lowers micro-crack density, and improves friction and wear performance. Simulation studies revealed the etching process and material removal mechanism of femtosecond laser on aluminum nitride ceramics. The width and depth of the melt pool in laser polishing samples were measured using optical cross-sectional micrographs and compared with simulated values, with relative tolerances of 3.23% and 12.5%, respectively.

## Figures and Tables

**Table 1:** Main components of aluminum nitride ceramic plate (wt%).

AlN	Y_2_O_3_	CaO	Al_2_O_3_	Si	Other
94.7%	4.8%	0.32%	0.008%	0.007%	0.165%

**Table 2:** Factor level design for response surface experiment.

Factor	Level		
	High	Medium	Low
laser power P/W	20	17.5	15
scanning speed ν/(mm/s)	1050	1000	950
pulse frequency *f*/kHz	325	300	275

**Table 3:** Simulation parameters for femtosecond laser polishing of aluminum nitride ceramic materials [26,27,28,,29,30,31,32,33].

Parameter (Unit)	Symbol	Value
Ambient temperature (K)	*T_*0*_*	300
Density (kg/m^3^)	ρ	3340
Laser spot radius (μm)	*r_*0*_*	10
Pulse duration (fs)	*tp*	500
Electron lattice coupling coefficient (W/m^3^·K)	*G*	2.11 × 10^11^
Bandgap energy (eV)	*E_g_*	6.2
Electron mobility (cm^2^/V·s)	*u_e_*	100
Original valence electron density (m^−3^)	*n_*0*_*	1.92 × 10^29^
Evaporation temperature (K)	*T_v_*	2723.15
Heat of evaporation (J/kg)	*H_s_*	2.27 × 10^7^
Electronic thermal conductivity (W/m·K)	*K_e_*	0.14
Lattice heat capacity coefficient (J/m^3^·K)	*K_l_*	1701
Thermal conductivity (W/m·K)	$C_{1}$	188
Electron heat capacity coefficient (J/m^3^·K^2^)	$C_{e}$	700
Laser reflectivity	*R*	0.3
Laser absorption coefficient (cm^−1^)	*α*	5.2 × 10^5^
Material constant for electron relaxation time (K^−2^·s^−1^)	*Ae*	1.2 × 10^−13^
Material constant for electron relaxation time (K^−1^·s^−1^)	*Bl*	3.5 × 10^−9^

**Table 4:** Design matrix and experimental results for response surface optimization.

No.	Parameters			Result	No.	Parameters			Result
	*P*/W	*v*/(mm/s)	*f*/kHz	*Ra*/μm		*P*/W	*v*/(mm/s)	*f*/kHz	*Ra*/μm
1	20	1050	325	0.782	10	15	1000	300	0.602
2	20	1050	275	0.721	11	17.5	1050	300	0.583
3	20	950	325	0.753	12	17.5	950	300	0.591
4	20	950	275	0.682	13	17.5	1000	325	0.572
5	15	1050	325	0.704	14	17.5	1000	275	0.563
6	15	1050	275	0.653	15	17.5	1000	300	0.548
7	15	950	325	0.735	16	17.5	1000	300	0.545
8	15	950	275	0.674	17	17.5	1000	300	0.541
9	20	1000	300	0.621					

**Table 5:** Analysis of variance for the surface roughness model.

Source	Sum of Squares	df	Mean Square	*F* Value	*p* Value	
Model	0.099	9	0.011	42.18	<0.0001	significant
A	3.648 × 10^−3^	1	3.648 × 10^−3^	13.94	0.0073	
B	6.400 × 10^−6^	1	6.400 × 10^−6^	0.024	0.8802	
C	6.401 × 10^−3^	1	6.401 × 10^−3^	24.45	0.0017	
AB	1.800 × 10^−3^	1	1.800 × 10^−3^	6.88	0.0343	
AC	5.000 × 10^−5^	1	5.000 × 10^−5^	0.19	0.6752	
BC	5.000 × 10^−5^	1	5.000 × 10^−5^	0.19	0.6752	
A^2^	0.018	1	0.018	67.54	<0.0001	
B^2^	8.623 × 10^−3^	1	8.623 × 10^−3^	32.94	0.0007	
C^2^	3.714 × 10^−3^	1	3.714 × 10^−3^	14.19	0.0070	
Residual	1.832 × 10^−3^	7	2.618 × 10^−4^			
*Lack of Fit*	1.800 × 10^−3^	5	3.599 × 10^−4^	22.04	0.0440	not significant
*Pure Error*	3.267 × 10^−5^	2	1.633 × 10^−5^			
R^2^ = 0.98189341080968			R^2^_adj_ = 0.95861351042213		R^2^_pre_ = 0.87284828300123	

Lack of Fit tests whether the chosen regression model adequately describes the observed data. Pure Error is estimated from replicated experimental data and represents the inherent variability that cannot be explained by any model.

## Data Availability

The original contributions presented in this study are included in the article. Further inquiries can be directed to the corresponding author.
